# *Cryptosporidium* Infection During Pregnancy and Effects on Pregnancy Outcomes in Israel

**DOI:** 10.3390/microorganisms12122572

**Published:** 2024-12-13

**Authors:** Shirley Shapiro Ben David, Olga Snitser, Miriam Parizade, Noam Orvieto, Yaacov Segal, Limor Adler

**Affiliations:** 1 Health Division, Maccabi Healthcare Services, Tel Aviv 6812509, Israel; 2 Family Medicine Department, Faculty of Medical & Health Science, Tel Aviv University, Tel Aviv 6997801, Israel; 3 National Mega Laboratories, Maccabi Healthcare Services, Mevo Carmel 2069236, Israel; 4 Maccabi National Laboratories, Mega Laboratory, Maccabi Healthcare Services, Rehovot 7670309, Israel

**Keywords:** *Cryptosporidium*, pregnancy, infection, diarrhea, outcome

## Abstract

*Cryptosporidium* is a parasite that typically causes self-limited gastroenteritis. Little is known about the course of infection and its impact during pregnancy. This retrospective cohort study conducted in Israel assessed the effects of *Cryptosporidium* infection on pregnancy and obstetrical outcomes. The study population included pregnant women with a positive PCR stool test for *Cryptosporidium* and a control group of pregnant women with negative tests, matched at a 3:1 ratio based on age, gestational week, and sector. Their medical records were retrieved for symptoms, treatments, pregnancy termination, gestational age, birth weight, Apgar score, and head circumference. Fisher’s exact or chi-squared tests were used to determine significance. Between January 2020 and December 2023, 2512 pregnant women underwent PCR stool testing. Of these, 93 were positive for *Cryptosporidium*, mostly in 2022. Their median age was 31 (range: 23–42 years), and 77.4% were from medium–high socioeconomic status. The most common symptom was diarrhea (90/93, 96.7%). The infection was self-limiting in all cases, with none requiring specific treatment. No significant differences were found in miscarriage rate, delivery week, birth weight, Apgar score, or head circumference compared to the controls. This study illuminates the clinical course of *Cryptosporidium* infection in pregnant women, emphasizing a generally favorable outcome without the necessity for therapeutic intervention.

## 1. Introduction

*Cryptosporidium*, an intracellular protozoan apicomplexan parasite, can lead to gastrointestinal infections in humans and animals and infect various vertebrate hosts, including birds, reptiles, and mammals [1,2]. In humans, the majority of cryptosporidiosis cases are caused by *C. hominis* and *C. parvum* [3].

*Cryptosporidium* is primarily transmitted through fecal–oral contamination, including ingesting contaminated water or food or through direct contact with infected individuals or animals [2]. Respiratory infections have also been reported [4]. *Cryptosporidium* undergoes a complete life cycle in a single host, comprising multiple rounds of asexual division, followed by subsequent sexual division and fertilization, leading to the formation of oocysts [5,6]. After ingestion, oocysts undergo excystation in the gastrointestinal (and sometimes respiratory) tract, releasing sporozoites that parasitize epithelial cells. The parasite multiplies within the host, producing thick-walled oocysts that are excreted into the environment and thin-walled oocysts that facilitate internal autoinfection [7].

*Cryptosporidium* spp. is well-suited for environmental transmission, mainly through waterborne and foodborne routes [6,7]. Key factors include its ability to infect multiple hosts, its worldwide distribution, and the shedding of large numbers of fully infective oocysts during acute infections [8,9]. Oocysts are small (4–6 µm), allowing them to bypass filtration systems, and are highly robust, with the ability to survive months in cool, moist conditions and resist chlorination [10,11]. Environmental transmission is further driven by their high survival rate in water (over 24 months at 20 °C), resistance to disinfection, low infectious dose, and low host specificity [12,13]. Consequently, *Cryptosporidium* poses a significant cause of waterborne outbreaks worldwide [13,14,15,16].

The transmission of *Cryptosporidium* is significantly impacted by socio-environmental factors, particularly poor sanitation [17,18,19,20]. Additionally, high population density can accelerate the circulation of pathogens, and heavy rainfall often washes *Cryptosporidium* oocysts from contaminated feces in watersheds into surface water sources, heightening the risk of water treatment failures [21,22,23]. To mitigate these risks, preventative measures such as maintaining proper hygiene and adopting robust water management systems are essential, especially during the onset of rainy seasons when contamination risks are elevated.

Cryptosporidiosis, caused by the *Cryptosporidium* parasite, is a gastrointestinal infection with a significant global cause of diarrhea in children and adults [6,24]. Although *Cryptosporidium* infections cause acute self-limiting gastroenteritis in immunocompetent individuals, chronic and life-threatening diarrheal disease may develop in immunocompromised individuals. Indeed, in certain immunocompromised patients, including those with AIDS or severe combined immunodeficiency syndrome, the disease may be very severe, prolonged, and life-threatening [25,26,27].

Little is known about the symptoms and prognosis of *Cryptosporidium* during pregnancy and the obstetrical outcomes [28,29]. Certain infections may pose an increased risk for pregnant women due to changes in immunity, including decreased cellular immune responses to selected antigens [30,31]. This makes them potentially more vulnerable to severe symptoms and complications. Cryptosporidiosis in pregnancy raises concerns about the potential adverse outcomes to women and fetuses. Indeed, pregnant women may experience more intense symptoms, including prolonged diarrhea, which can lead to dehydration, nutritional deficiencies, and electrolyte imbalances. Treatment options are limited during pregnancy as many antiparasitic medications are contraindicated due to potential risks to the fetus [32]. Therefore, management often focuses on supportive care, including hydration and electrolyte balance maintenance, to alleviate symptoms and prevent complications [24].

The incidence of *Cryptosporidium* in Israel is usually rare [33]. However, in 2022, a notable rise in the incidence of cryptosporidiosis was observed in Israel, including in pregnant women. This study aimed to evaluate any influence of *Cryptosporidium* infection on pregnancy course and obstetrical outcomes.

## 2. Materials and Methods

### 2.1. Study Design and Setting

A retrospective case-control study was conducted in the Maccabi Healthcare Services (MHS), Israel’s second-largest health maintenance organization (HMO). The MHS offers outpatient care for more than 2.6 million members nationwide, accounting for about a fourth of Israel’s population. The MHS’ central data repository retains patient demographic data, physician data, and laboratory results using each patient’s unique national identification number. Laboratory tests, including stool tests, are conducted in centralized laboratories. All healthcare providers within the MHS utilize a unified electronic medical record (EMR) system through which all laboratory test results are reported.

### 2.2. Microbiological Technique

Fecal specimens were self-collected by patients and transported to the laboratory using commercial Cary-Blair transport media (Fecal Swab, Copan, Brescia, Italy). In the laboratory, specimens were vortexed vigorously for 2 min, then centrifuged at 900× *g* for 30 s, and the DNA was extracted using STARMag 96 × 4 Universal Cartridge Kit (Seegene, Seoul, Republic of Korea) on the Microlab STARlet IVD (Hamilton, Hercules, CA, USA) platform. *Cryptosporidium* spp. DNA was amplified and detected using the multiplex real-time PCR Allplex^TM^ GI-Parasite Assay kit (Seegene, Seoul, Republic of Korea) on the CFX96^TM^ real-time PCR detection system (Bio-Rad, Bonaduz, Switzerland). The PCR primer was targeted at the 18S *Cryptosporidium* gene. Seegene viewer software (versions 3.28.000 and 3.29.000, Seegene, Seoul, Republic of Korea) was used for data analysis. The whole process was controlled using an internal control within the clinical specimens. The control within the clinical specimens was a non-human plasmid that went through the entire DNA extraction procedure. The control was part of the Allplex^TM^ GI-Parasite Assay kit (Seegene, Seoul, Republic of Korea). The real-time PCR runs were validated by the accompanying positive and negative controls.

### 2.3. Participants

The study population consisted of pregnant women who tested positive for *Cryptosporidium* in stool samples. The matched control group consisted of pregnant women who were referred for PCR stool tests, with results negative for *Cryptosporidium* or any other pathogen. Matching in the ratio of 1:3 was based on age, gestational stage (week) at the time of testing, and sector (Arab, General Jewish, and Ultra-Orthodox Jewish).

### 2.4. Variables

Medical record data were collected for demographics, immunosuppression status, and comorbidities, including inflammatory bowel disease, diabetes, and kidney disease, based on the MHS’ registries [34], as well as antibiotic and anti-parasitic treatments, and pregnancy and obstetrical data. Details on triggers and symptoms, according to the doctor’s report and the course of pregnancy, were explicitly sought from the medical records.

Outcome parameters included live birth/miscarriage, gestational age—term/preterm (term defined as 37 weeks or more)—type of delivery, birth weight, head circumference, and Apgar score. The Apgar score is a quick and standardized method to assess a newborn’s clinical status and the need for intervention immediately after birth [35]. It assesses five components—color, heart rate, reflexes, muscle tone, and respiration—each assigned a score from 0 to 2. The score is recorded at 1 and 5 min after birth. Statistical significance was determined using chi-squared tests for categorical variables and the Mann–Whitney test for continuous variables. All the statistical tests were two-sided, and *p*-value < 0.05 was considered statistically significant. Statistical analysis was performed with SPSS version 28.

The study was conducted in accordance with the Declaration of Helsinki and approved by the Institutional Review Board (or Ethics Committee) of the Maccabi Healthcare Services (MHS-22-0070) on 7 August 2022.

## 3. Results

### 3.1. Participants

**Figure 1:**
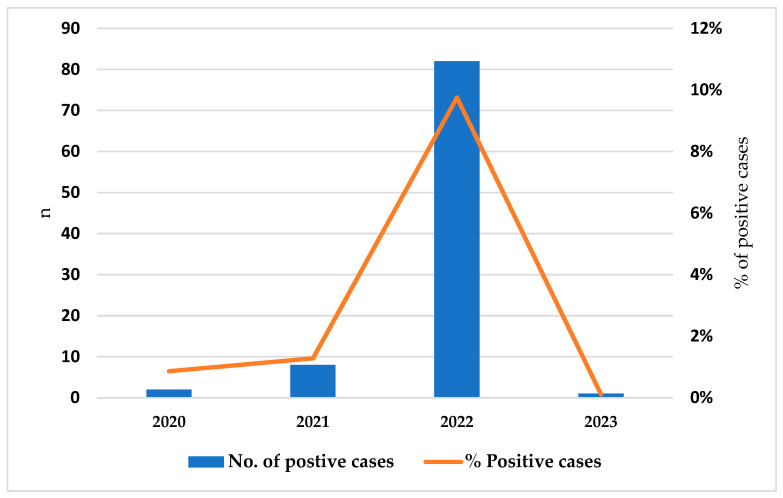
Epidemic curve of the number and percentage of pregnant women who performed stool tests and tested positive for *Cryptosporidium* spp. between January 2020 and December 2023: Total number of women was 2514; total number of positive cases was 537.

Between January 2020 and December 2023, 93 stool samples from pregnant women tested positive for *Cryptosporidium*. The highest incidence occurred in 2022, with a peak observed during the summer months (third quarter), as shown in Figure 1. The baseline characteristics of the patients studied are summarized in Table 1. Their median age was 31, ranging from 23 to 42 years old, with medium–high socioeconomic status (72, 77.4%). Women were generally healthy and without comorbidities. All were negative for HIV. The tests were performed in 31 cases (33.3%) during the first trimester, 38 cases (40.9%) during the second, and 24 cases (25.8%) during the third. Most pregnancies (89/93) were naturally conceived, and 11.8% (10/93) were considered high risk (due to chronic kidney disease, hypertension, gastric bypass, inflammatory bowel disease, recurrent urinary tract infection, intrauterine growth retardation, and gestational diabetes).

### 3.2. Descriptive Data

**Figure 2:**
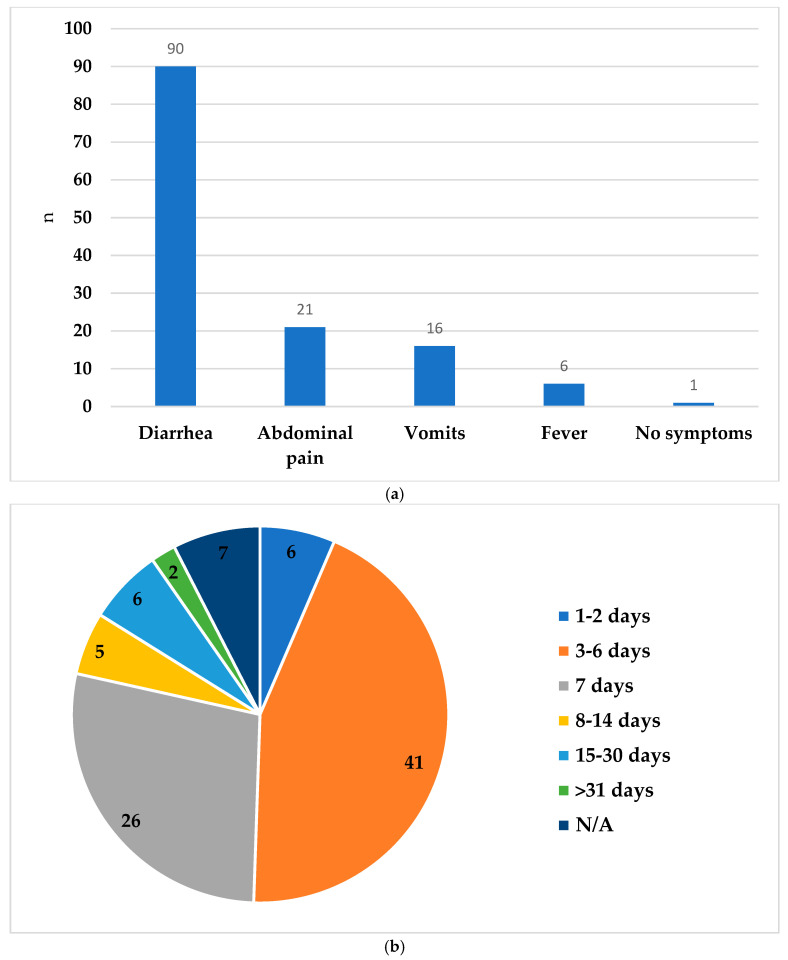
Clinical symptoms and duration of cryptosporidiosis in pregnant women (**a**) Symptoms of pregnant women tested positive for *Cryptosporidium*; (**b**) Duration of symptoms in days. N/A—Not available.

Information about the source of infection was available for only 45 of the 93 cases. Among these, 35 cases were linked to a household with gastrointestinal symptoms, predominantly children (33 of 35 cases), and 10 cases were associated with outdoor activities, primarily swimming. The most common symptom was diarrhea (90/93, 96.7%), followed by abdominal pain (21/93, 22.6%), nausea or vomiting (16/93, 17.2%), and fever (6/93, 6.4%) (Figure 2a). The symptoms lasted for up to a week in most cases (73 of 93, 78.5%), with only two cases experiencing symptoms for over a month (Figure 2b). All infections were self-limiting. Less than one-third of the women (30) needed intravenous fluid administration. One woman was treated with azithromycin, one with metronidazole, and one with amoxicillin. Only three women were admitted within a week before or after the positive stool test, with stays lasting less than three days.

### 3.3. Outcome Data

A comparison of the various maternal and neonatal outcomes between the two groups—*Cryptosporidium*-positive and *Cryptosporidium*-negative individuals—was performed, as shown in Table 2. The rate of miscarriage in the two groups was similar (3.2% and 4.1%, respectively, NS). Most had a term delivery (93.5% and 90.7%). No significant differences between the two groups were found for the neonatal outcome, including birth weight (3275 (388.5) grams vs. 3218.5 (636.7) grams, NS), head circumference (34.1 (1.3) centimeters vs. 33.6 (5.0) centimeters, NS), Apgar scores, and gestational age (38.8 (1.7) weeks vs. 38.9 (1.4) weeks, NS).

## 4. Discussion

To our knowledge, this is the first study to evaluate the impact of *Cryptosporidium* infection during pregnancy. Our findings reveal that infected pregnant women predominantly experienced self-limiting diarrhea. Notably, there were no significant adverse effects on obstetrical outcomes such as preterm birth, low birth weight, or any other complications. These results provide valuable insights into the clinical course of *Cryptosporidium* infection in pregnant women.

*Cryptosporidium* is a rare parasitic infection, usually with sporadic cases in a year. In 2022, Israel experienced a surge in *Cryptosporidium* cases, with the peak occurring during the summer months. The absolute number of cases and the percentage of positive stool tests increased. The cause of this surge remains unknown. One suggested explanation was an overall increase in the number of PCR stool tests conducted. However, this seems unlikely, as the percentage of positive cases declined again in 2023. Considering that some symptomatic cases did not undergo stool tests, the actual incidence of these conditions in pregnant women and the general population may have been underreported. As a result, the exact number of cases in 2022 could be much higher than currently documented. Interestingly, the background of IBD was four times higher in the control group than in the infected group, likely due to the large number of tests conducted in the control group, which revealed the non-infectious causes for diarrhea [36]. This cohort revealed that the vast majority of infected pregnant women experienced self-limiting gastrointestinal symptoms, primarily diarrhea. This finding aligns with the previous literature, which describes *Cryptosporidium* as typically causing mild, self-limiting gastrointestinal illness in immunocompetent individuals, including pregnant women [28]. This suggests that the partial immune suppression associated with pregnancy does not significantly alter the clinical course of cryptosporidiosis. Notably, none of the women in our study required specific treatment, such as nitazoxanide, an FDA pregnancy Category B drug, indicating that the infection resolved without pharmacological intervention, unlike other parasites [31,37,38,39].

The comparative analysis with matched controls revealed no statistically significant differences in key obstetrical outcomes, including miscarriage rate, delivery week, birth weight, Apgar score, and head circumference. These findings are reassuring for both healthcare providers and pregnant women, suggesting that *Cryptosporidium* infection, while unpleasant, does not pose a significant threat to fetal development or pregnancy progression.

The study’s main strength is its comprehensive approach, encompassing a wide range of obstetrical outcomes and a sizeable cohort of pregnant women. The use of matched controls enhances the validity of the findings by accounting for potential confounding factors such as age, stage of pregnancy, and socioeconomic status. However, there are limitations to consider. The study’s retrospective nature relies on the accuracy and completeness of medical records, which may introduce some biases. In addition, the study was conducted in a developed country and may not be generalized to developing countries. *Cryptosporidium* infection is more common in developing areas, due to inadequate access to clean drinking water and sanitation facilities [25,40]. Lastly, while no immediate adverse outcomes were observed, the long-term effects of *Cryptosporidium* infection during pregnancy remain unclear. Further longitudinal studies are needed to assess the potential delayed impacts on maternal and fetal health.

## 5. Conclusions

In conclusion, this study contributes to our understanding of *Cryptosporidium* infection in pregnant women, highlighting a favorable prognosis without significant obstetrical consequences. While the immediate outcomes appear benign, ongoing research is essential to elucidate any long-term effects and to optimize preventive strategies for this vulnerable population.

## Figures and Tables

**Table 1:** Baseline patient characteristics and underlying conditions.

	*Cryptosporidium* Positive (n = 93)	*Cryptosporidium* Negative (n = 291)	*p* Value
Age (mother)	31.0 (4.4)	30.3 (4.3)	0.231
Sector (n, %)			
Arab	3 (3.2)	8 (2.7)	
Ultra-Orthodox Jewish	17 (18.3)	34 (11.7)	0.269
General Jewish	73 (78.5)	249 (85.6)	
Socioeconomic status (n, %)			
Low	21 (22.6)	54 (18.6)	
Middle	36 (38.7)	127 (43.6)	0.603
High	36 (38.7)	110 (37.8)	
Co-morbidities (n, %)			
Inflammatory Bowel Disease	2 (2.1)	24 (8.2)	0.055
Hypertension	1 (1)	3 (1)	>0.999
Diabetes	1 (1)	1 (0.3)	0.426
Kidney disease	2 (2.1)	3 (1)	0.598
HIV, positive	0	0	
Naturally conceived pregnancy (n, %)	89 (95.7)	255 (87.6)	0.031
High-risk pregnancy (n)	10 (10.8)	36 (15)	0.378
Gestational age at time of test (mean, SD; week)	18.9 (10.2)	18.8 (10.3)	0.943

**Table 2:** Obstetrical outcomes among pregnant women infected with *Cryptosporidium* and their matched control.

Outcome	*Cryptosporidium* Positive (n = 93)	*Cryptosporidium* Negative (n = 291)	*p*-Value
Miscarriages n (%)	3 (3.2)	12 (4.1)	0.773
Term Delivery n (%)	87 (93.5)	264 (90.7)	0.525
Vaginal delivery n (%)	75 (80.6)	228 (78.4)	0.863
Birth weight, grams, mean (SD)	3275.0 (388.5)	3218.5 (636.7)	0.992
Head circumstance, centimeters, mean (SD)	34.1 (1.3)	33.6 (5.0)	0.346
Apgar (1), median	8.9 (0.5)	8.9 (0.4)	0.824
Apgar (5), median	9.8 (0.4)	9.9 (0.5)	0.103
Gestation age at birth, week, mean (SD)	38.8 (1.7)	38.9 (1.4)	0.831

## Data Availability

The data supporting this study are available from the corresponding author, but restrictions apply to the availability of such information. It was used under a license for the current study and is not publicly available. Data are, however, available from the authors upon reasonable request and with permission of the local ethics committee of MHS.
